# Association between systemic inflammation response index trajectories and carotid atherosclerosis progression

**DOI:** 10.3389/fendo.2025.1676493

**Published:** 2025-10-14

**Authors:** Ningning You, Jing Su, Yi Chen, Jun Chen, Jinshun Zhang

**Affiliations:** ^1^ Department of Gastroenterology, Taizhou Hospital of Zhejiang Province affiliated to Wenzhou Medical University, Taizhou, Zhejiang, China; ^2^ Hematology Laboratory, Suqian First People’s Hospital Affiliated to Nanjing Medical University, Suqian, Jiangsu, China; ^3^ Health Management Center, Suqian First People’s Hospital Affiliated to Nanjing Medical University, Suqian, Jiangsu, China; ^4^ Home Ward, Taizhou Hospital of Zhejiang Province affiliated to Wenzhou Medical University, Taizhou, Zhejiang, China; ^5^ Health Management Center, Taizhou Hospital of Zhejiang Province affiliated to Wenzhou Medical University, Taizhou, Zhejiang, China; ^6^ Shaoxing University, Shaoxing, Zhejiang, China

**Keywords:** systemic inflammation response index, carotid atherosclerosis, progression, longitudinal study, trajectories

## Abstract

**Background:**

The systemic inflammation response index (SIRI) has emerged as a promising inflammatory biomarker linked to the onset and progression of cardiovascular disease (CVD). However, the association between initial and long-term trajectories of the SIRI index and carotid atherosclerosis (CAS) progression remains unexplored.

**Methods:**

This longitudinal retrospective cohort study encompassed 11,623 adults undergoing multiple general health checks at Taizhou Hospital of Zhejiang Province from January 2017 to September 2024. SIRI values were derived using the formula: neutrophil count × monocyte count/lymphocyte count. To assess SIRI trends over time, latent class trajectory modeling was utilized. Hazard ratios (HRs) and 95% confidence intervals (CIs) for both the initial and trajectories of the SIRI index were determined through univariate and multivariate Cox proportional hazards analyses. Restricted cubic splines evaluated potential nonlinear associations between SIRI and CAS risk.

**Results:**

**O**ver a median follow-up of 2,043 days, 2,460 individuals experienced progression of CAS. After adjusting for conventional CVD risk factors, a 1-standard deviation (SD) rise in SIRI was linked to a 12% elevated risk of CAS progression (HR = 1.121, 95% CI 1.035–1.213). Comparable findings were noted when SIRI was stratified into quartiles. Participants were classified into three trajectory groups: low-stable, middle-stable, and high-stable. Following multivariate adjustments, the high-stable group exhibited a 1.166-fold increased risk of CAS progression (95% CI 1.021–1.333).

**Conclusions:**

Elevated initial SIRI levels and a high-stable trajectory were associated with an increased risk of CAS progression. Tracking SIRI trends over time may help identify individuals at heightened risk, enabling more focused prevention and treatment strategies.

## 1 Introduction

Cardiovascular disease (CVD) is the leading factor behind disability and premature mortality globally, posing a major economic and healthcare burden. Based on the Global Burden of Disease Study 2019, the number of prevalent cases of total CVD nearly doubled from 271 million in 1990 to 523 million in 2019 (1). Atherosclerosis, a major pathological process in most cardiovascular diseases, can begin as early as childhood and progress asymptomatically for decades (2). Early detection of arterial disease in seemingly healthy individuals often focuses on the peripheral arteries, particularly the carotid arteries (3). The progression of carotid atherosclerosis (CAS) results from a complex interaction of factors, including lipid metabolism, hemodynamic stress, and systemic inflammation (4). Among these, inflammation has emerged as a critical driver of atherosclerotic plaque formation and destabilization. Recent studies have highlighted the potential of systemic inflammatory biomarkers in predicting plaque progression and cardiovascular outcomes (5–8).

Inflammatory markers such as platelet and lymphocyte counts, along with ratios like neutrophil-to-lymphocyte (NLR) and platelet-to-lymphocyte (PLR), have been associated with an increased risk of adverse cardiovascular events and progression of coronary artery disease (9–14). Other leukocyte indicators, such as monocyte count and platelet count, may also be correlated with the presence of CAS (15, 16). The relatively novel index, the SIRI, integrates neutrophil, monocyte, and lymphocyte counts and has initially been used to predict survival in cancer patients (17); Despite being considered a novel inflammatory biomarker, SIRI is more comprehensive, easily accessible, and has been broadly validated across multiple studies (18–20). It effectively reflects the inflammatory status of the human body. Besides, research has shown that SIRI may outperform classic inflammatory indicators such as the NLR, PLR, and Monocyte-to-Lymphocyte Ratio (MLR) in predicting stroke prognosis (18). For instance, Zhang et al. (18)utilized Receiver Operating Characteristic (ROC) analysis to demonstrate that SIRI had better predictive accuracy for stroke outcomes than PLR, NLR, or MLR. Similarly, among patients with acute coronary syndrome, SIRI has been identified as a more reliable inflammatory biomarker than NLR and MLR (20).

Currently, an increasing number of research studies have revealed the association between SIRI and CAS (21–24). However, most studies have been cross-sectional and have not provided an in-depth exploration of the relationship between the dynamic changes (trajectories) of SIRI and the progression of CAS. In recent years, trajectory models - such as latent variable growth models and mixed effects models - have gained widespread application in studying the dynamic changes of biomarkers and their relationship with disease progression (25, 26). Nevertheless, the relationship between SIRI trajectories and the progression of CAS remains unexplored using these methods, which offer a more nuanced understanding of temporal trends and potential causal influences.

Based on this, we proposed that fluctuations in systemic inflammation could play a role in the progression of CAS. Using a large longitudinal single-center cohort of Chinese individuals, this study aimed to examine the association of both baseline SIRI levels and their long-term trajectories with CAS progression.

## 2 Method

### 2.1 Study design and population

**Figure 1:**
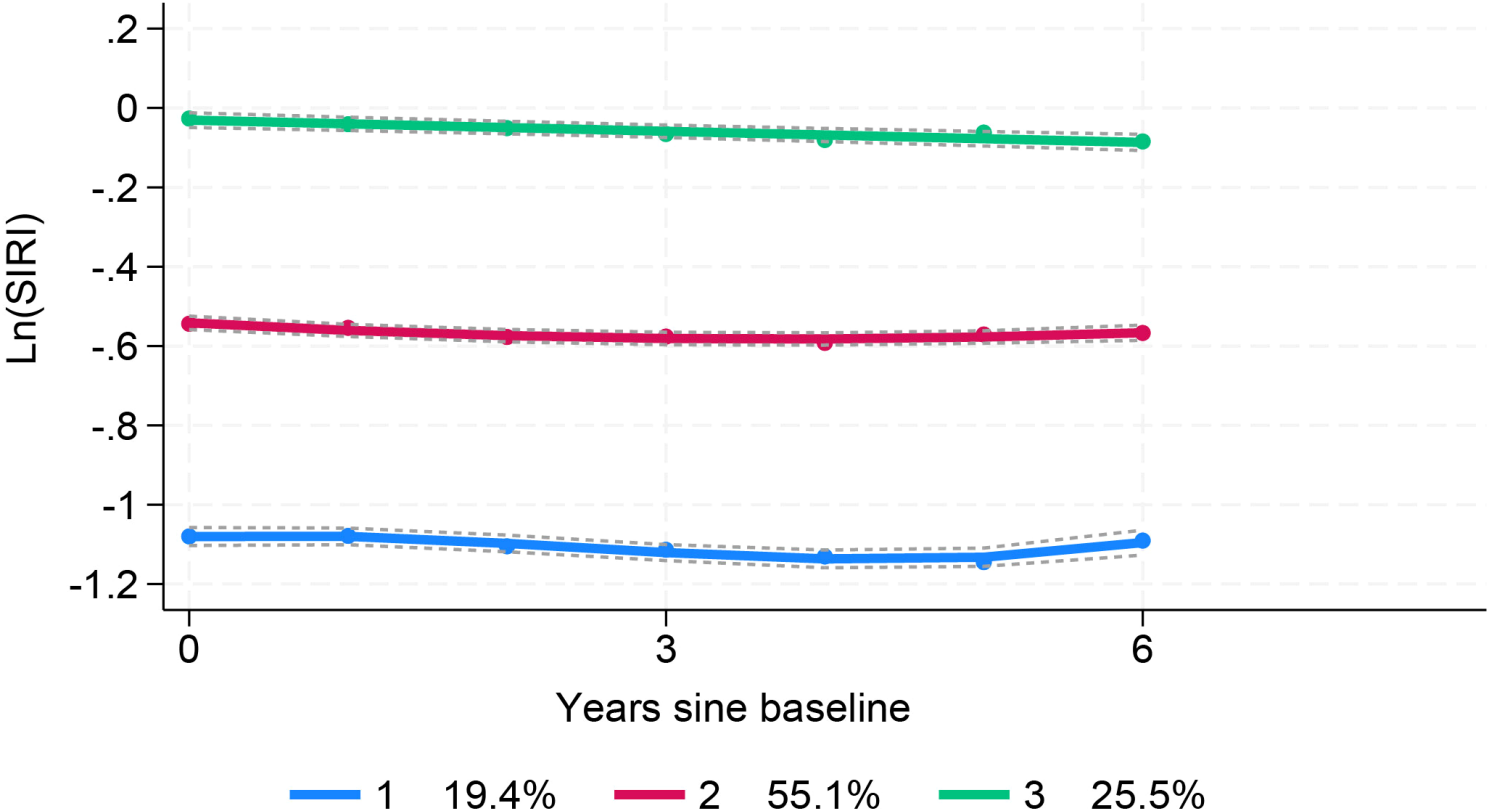
Flow diagram of study selection for individuals with health examination who performed the carotid ultrasonograph and blood tests.

This population-based, retrospective longitudinal cohort study utilized data from routine health examinations conducted at Taizhou Hospital of Zhejiang Province. Between January 2017 to September 2024, a total of 33425 participants aged 18 years or older, who had completed at least two general medical check-ups, were initially enrolled (27). Exclusion criteria included: (1) A recent history of viral or bacterial infections (n=925); (2) Individuals with chronic autoimmune diseases, hematologic disorders, liver cirrhosis, or oncologic malignancies (n=658); (3) Absence of carotid ultrasonography data (n=10967); (4) With existing carotid artery plaques (n=7369); (5) Lack of blood routine data (n=1883). After exclusions, 11623 individuals were included in the baseline analysis. The same cohort of 11,623 participants was also used for trajectory analysis. A detailed flowchart of the study is presented in **Figure 1**. The study protocol was reviewed and approved by Ethics Committee of Taizhou Hospital (K20220790).

### 2.2 Characteristics and definition

Data on demographic characteristics and medical history were collected by trained interviewers using a standardized questionnaire. Diabetes was identified as fasting blood glucose (FBG) ≥7.0 mmol/L during the examination or self-reported physician diagnosis of diabetes (28). Systolic (SBP) and diastolic blood pressure (DBP) were measured as the average of three seated readings using an automated blood pressure monitor. Hypertension was defined as SBP ≥140 mmHg or DBP ≥90 mmHg, current use of antihypertensive drugs, or a self-reported diagnosis of hypertension. Body mass index (BMI) was computed as weight (kg)/height (m)^2^. *Helicobacter pylori* (*H. pylori*) infection was assessed using 13C or 14C urease breath tests (29). Biochemical parameters analyzed included FBG, total cholesterol (TC), triglycerides (TG), low-density lipoprotein cholesterol (LDL-C), and high-density lipoprotein cholesterol (HDL-C) (30). Peripheral blood samples were processed by the Clinical Laboratory Department of Taizhou Hospital of Zhejiang Province, which holds a laboratory accreditation certificate. Fasting blood samples were collected in the morning, and biochemical analyses were performed using a Beckman Coulter platform (Beckman Coulter Inc., Brea, CA, USA) with commercially available assay kits.

### 2.3 Carotid ultrasonography and study outcome

Bilateral carotid artery assessments were performed manually by certified and experienced ultrasound specialists from Taizhou Hospital of Zhejiang Province, who were blinded to the study details. The examinations utilized a GE^®^ Vivid i/E95 high-resolution ultrasound system equipped with a 7.5–12 MHz phased array probe. Abnormal carotid intima-media thickness (cIMT) was defined as a maximum cIMT value ≥0.9 mm, measured as the greatest distance between the lumen-intima and media-adventitia interfaces. Carotid plaque was identified as cIMT ≥1.5 mm, a focal structure protruding into the arterial lumen by ≥0.5 mm, or ≥50% of the surrounding cIMT value. Furthermore, CAS progression was characterized by the development of new carotid stenosis, plaque, or increased cIMT during follow-up compared to baseline (**Supplementary Figure 1**). For participants with both carotid plaque and cIMT, baseline and follow-up results were determined based on the more superior manifestations (i.e., carotid plaques) (31–34).

### 2.4 Systemic inflammation response index (SIRI, SII, LMR, PLR, NLR)

Systemic inflammation response index derived from complete blood counts, such as the SIRI, SII, LMR, PLR, and NLR, have been widely used to predict risk and prognosis in various diseases (35–37). In this study, we aimed to thoroughly elucidate the relationship between systemic inflammatory biomarkers and carotid atherosclerosis. To this end, we calculated the SIRI, SII, LMR, PLR, and NLR using the following formulas: SIRI = neutrophil count × monocyte count/lymphocyte count, SII = platelet counts × neutrophil counts/lymphocyte counts, LMR = lymphocyte counts/monocyte counts, PLR = platelet counts/lymphocyte counts, NLR = neutrophil counts/lymphocyte counts.

### 2.5 Statistical analyses

Continuous variables are presented as mean ± standard deviation, while categorical variables are expressed as frequency (percentage). Comparisons of continuous variables were conducted by using Mann–Whitney U tests or Kruskal–Wallis H-tests (two or more independent samples), and comparisons of categorical variables were analyzed using the chi-squared test or Fisher’s exact test. Given the skewed distributions of the SIRI, SII, LMR, PLR, and NLR, natural logarithm (ln) transformation were applied to approximate normal distributions, and the values were categorized into quartiles (Q1, Q2, Q3, and Q4). The Cox proportional hazards regression model was used to access the association between baseline SIRI index quartiles (or per standard deviation change) and CAS progression, adjusting for potential confounders such as age, SBP, FPG, and BMI. The cumulative incidence of CAS progression across SIRI quartiles was visualized using Kaplan-Meier survival curves, with significance determined by log-rank tests. Additionally, we utilized restricted cubic splines within the Cox model framework to examine potential nonlinear dose-response relationships between SIRI values and CAS risk.

Latent class trajectory modeling (LCTM) was used to characterize long-term trends in SIRI. This method identifies homogeneous subgroups within heterogeneous longitudinal data by grouping participants with similar SIRI trajectories. The optimal number of trajectory classes was determined based on: (1) the lowest Bayesian Information Criteria (BIC) while maintaining clinical relevance and model parsimony; (2) an average probability of assignments above 70% for all latent classes; and (3) each class comprising at least 2% of the study population (26). We fitted models with two to four classes. Model selection was based on a combination of statistical criteria and clinical interpretability. The interpretability required that each class represented a substantively distinct and clinically meaningful pattern and that all classes contained a sufficient proportion of the sample (>2%). After comparing all models, the 3-class solution was chosen as it offered the optimal balance of statistical fit and parsimony. Trajectory class characteristics were compared using ANOVA or Kruskal-Wallis H-tests for continuous variables and chi-square tests for categorical variables. The association between trajectory classes and CAS progression was evaluated using Cox proportional hazards regression, with follow-up time as the time scale.

All of the statistical analyses were conducted using Stata version 18.0 (Stata Corp, College Station, TX, USA), R software (version 4.1.3), and IBM SPSS software (version 23.0, SPSS Inc., Chicago, IL). A two-tailed p-value <0.05 was considered statistically significant.

## 3 Results

### 3.1 Baseline characteristics according to SIRI index quartiles

This study involved 11623 eligible participants with a median age was 47 (39–54) years, of whom 7,658 (65.9%) were male. The median Ln(SIRI) index was 0.6 (0.42–0.83). Over a median follow-up period of 2043 days (IQR: 1428–2204 days), 2460 (21.2%) participants met the study outcome. Participants were categorized into four groups based on the SIRI index levels (**Table 1**). Individuals in higher Ln(SIRI) quartiles tended to be younger, male, and had a higher BMI, as well as a greater prevalence of hypertension, smoking, alcohol consumption, and *H. pylori* infection compared to those in the lowest quartile. Additionally, SBP, DBP, TG, TC, SIRI, SII index, PLR, and NLR index showed positive correlations with increasing Ln(SIRI) quartiles. In contrast, HDL-C levels and LMR index exhibited negative correlations (all *p* for trend<0.001). These results suggested that elevated SIRI levels are linked to a higher burden of cardiometabolic risk factors in the study population.

**Table 1:** Baseline characteristics of study participants according to quartiles of SIRI index.

Characteristics	Quartiles of ln (SIRI) index				P for trend
	Q1 (< -0.86)	Q2 (-0.86~ -0.51)	Q3 (-0.51~ -0.18)	Q4 (> -0.18)	
n	2929	3050	2739	2905	
Age (years)	48 (40-55)	47 (39-54)	47 (39-53)	47 (40-54)	.017
Male, n (%)	1610 (55%)	1963 (64.4%)	1924 (70.2%)	2161 (74.4%)	.000
BMI (kg/m2)	23.52 (21.5-25.7)	24.08 (22.1-26.1)	24.32 (22.5-26.6)	24.54 (22.6-26.6)	.000
Diabetes mellitus, n (%)	52 (1.8%)	63 (2.1%)	57 (2.1%)	81 (2.8%)	0.055
Hypertension, n (%)	104 (3.6%)	143 (4.7%)	150 (5.5%)	169 (5.8%)	0.000
Alcohol consumption, n (%)	148 (5.1%)	205 (6.7%)	230 (8.4%)	251 (8.6%)	0.000
Smoking, n (%)	311 (10.6%)	422 (13.8%)	510 (18.6%)	669 (23.0%)	0.000
H. Pylori positive, n (%)	780 (10.3%)	799 (10.6%)	791 (10.5%)	855 (11.3%)	0.039
SBP (mmHg)	122 (112-133)	123 (113-135)	124 (114-136)	124 (115-136)	.000
DBP (mmHg)	74 (66-82)	75 (67-83)	76 (68-84)	76 (69-85)	.000
LDL (mmol/L)	2.6 (2.2-3.1)	2.6 (2.2-3.1)	2.6 (2.2-3.1)	2.6 (2.1-3.0)	0.04
HDL (mmol/L)	1.4 (1.3-1.7)	1.4 (1.3-1.7)	1.4 (1.2-1.5)	1.3 (1.2-1.5)	.000
TC (mmol/L)	5.0 (4.5-5.6)	5.0 (4.4-5.6)	4.9 (4.4-5.6)	4.9 (4.3-5.5)	.000
TG (mmol/L)	1.4 (0.9-2.1)	1.5 (1.0-2.2)	1.6 (1.1-2.4)	1.6 (1.1-2.5)	.000
FPG (mmol/L)	5.0 (4.7-5.4)	5.0 (4.7-5.4)	5.0 (4.6-5.4)	5.0 (4.6-5.4)	0.149
Leukocytes (1000 cell/mL)	5.2 (4.5-5.9)	5.8 (5.1-6.6)	6.4 (5.7-7.3)	7.5 (6.5-8.5)	.000
Neutrophil count (1000 cell/mL)	2.6 (2.2-3)	3.3 (2.9-3.7)	3.8 (3.4-4.3)	4.8 (4.1-5.5)	.000
Platelet count (1000 cell/mL)	225 (196-258)	235 (203-269)	240 (210-277)	246 (210-282)	.000
Lymphocyte count (1000 cell/mL)	2.1 (1.8-2.5)	2.1 (1.7-2.5)	2 (1.7-2.4)	2.0 (1.6-2.4)	.000
Monocyte count (1000 cell/mL)	0.3 (0.2-0.3)	0.3 (0.3-0.4)	0.4 (0.3-0.4)	0.5 (0.4-0.5)	.000
SIRI	0.3 (0.3-0.4)	0.5 (0.5-0.6)	0.7 (0.7-0.8)	1.1 (0.9-1.3)	.000
SII	273.7 (219.7-339.6)	367.1 (304.7-445.7)	441.5 (366.0-534.5)	594.8 (477.9-738.8)	.000
NLR	1.2 (1.0-1.4)	1.6 (1.4-1.8)	1.9 (1.6-2.1)	2.4 (2.1-2.9)	.000
PLR	106.3 (86.9-130.0)	113.9 (93.7-137.4)	116.8 (965.0-141.7)	125.0 (101.6-155.0)	.000
LMR	8 (7-9.5)	6.3 (5.7-7.3)	5.4 (4.8-6.0)	4.3 (3.6-5.0)	.000
CAS progression, n (%)	535 (18.3%)	628 (20.6%)	603 (22.0%)	694 (23.9%)	.000

### 3.2 Associations between baseline SIRI index and CAS progression

**Figure 2:**
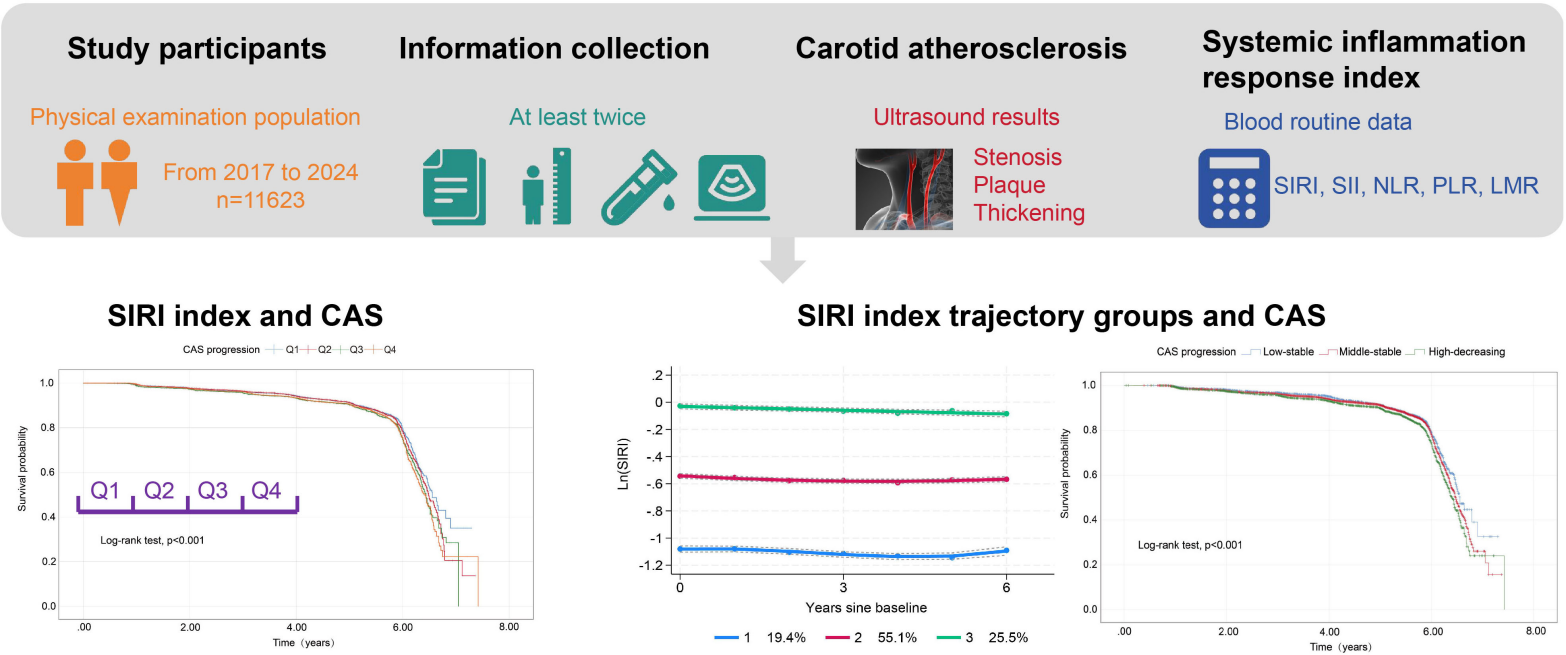
Kaplan–Meier survival analysis curves for CAS progression based on quartiles of baseline SIRI index. Ln(SIRI) index: Q1 (< -0.86),Q2 (-0.86 ~ -0.51),Q3 (-0.51 ~ -0.18), Q4(> -0.18). CAS, carotid atherosclerosis; SIRI, systemic inflammation response index.

As presented in **Table 1**, the risk of progression of CAS rose with increasing quartiles of the Ln(SIRI) index. In multivariate analyses treating the Ln(SIRI) index as a continuous variable, a 1-standard deviation (SD) increase in the Ln(SIRI) index was linked to a 12% higher risk of CAS progression (HR = 1.121, 95% CI 1.035–1.213, *p* = 0.005, as shown in **Table 2**). Similar patterns were observed when participants were stratified by Ln(SIRI) quartiles; Specifically, individuals in the highest Ln(SIRI) quartile exhibited the greatest risk of CAS progression across all adjusted models (all *p* < 0.05, **Table 2**). In the final model, the HRs with 95% CIs for CAS progression in the second, third, and fourth quartiles compared to the first quartile were 1.049 (95% CI 0.929–1.184), 1.212 (95% CI 1.072–1.371), and 1.186 (95% CI 1.052–1.337), respectively (**Table 2**). **Figure 2** illustrates the Kaplan–Meier survival curves for CAS progression by baseline Ln(SIRI) quartiles (log-rank test, *p* < 0.001). The RCS analysis revealed a nonlinear positive association between SIRI and CAS risk, with an inflection point at Ln(SIRI) = 0.35 (*p* < 0.001, **Supplementary Figure 1**).

**Table 2:** Hazard ratios (95% confidence intervals) of CAS progression by baseline Ln (SIRI) index.

Ln (SIRI) index	CAS progression (N)	Unadjusted HR (95%CI)	P value	Model 1 HR (95%CI)	P value	Model 2 HR (95%CI)	P value
Quartile1	535/2929	Reference		Reference		Reference	
Quartile2	628/3050	1.092 (0.970-1.230)	0.147	1.060 (0.939-1.196)	0.345	1.049 (0.929-1.184)	0.440
Quartile3	603/2739	1.126 (1.088-1.383)	0.001	1.233 (1.091-1.394)	0.001	1.212 (1.072-1.371)	0.002
Quartile4	694/2905	1.279 (1.138-1.437)	0.000	1.209 (1.074-1.362)	0.002	1.186 (1.052-1.337)	0.005
Per 1 SD	2460/11623	1.189 (1.10-1.285)	0.000	1.136 (1.050-1.229)	0.001	1.121 (1.035-1.213)	0.005

Model1: Adjusted for age, SBP and FPG;

Model2: Adjusted for age, SBP, FPG and BMI.

### 3.3 Baseline characteristics according to SIRI index trajectories

**Figure 3:**
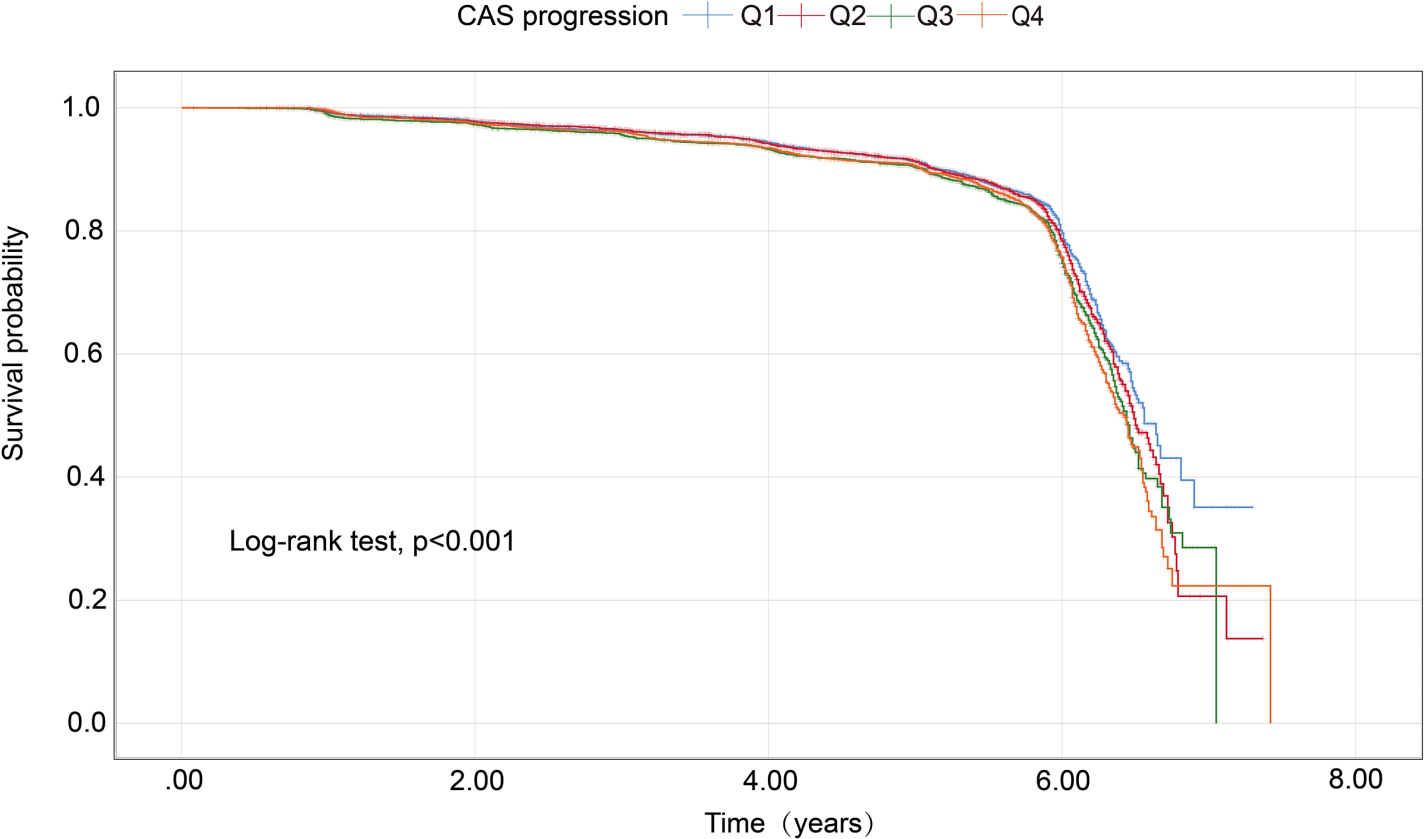
SIRI index trajectory groups and percentage of the participants in the grou SIRI, systemic inflammation response index.

**Figure 4:**
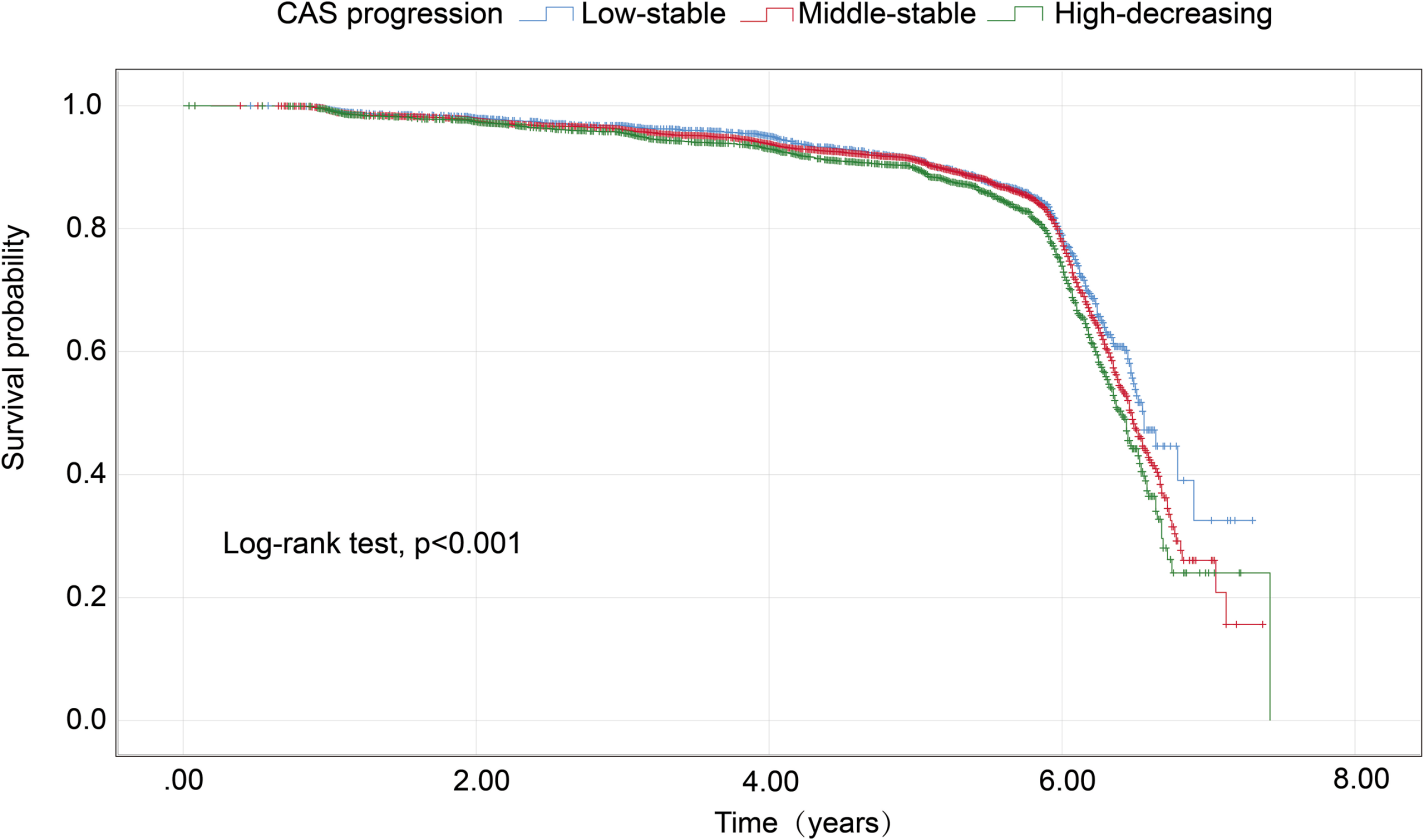
Kaplan–Meier survival analysis curves for CAS progression based on trajectories of the SIRI index. CAS, carotid atherosclerosis; SIRI, systemic inflammation response index.

Trajectory analysis included all 11,623 participants (**Figure 1**). The optimal 3-group trajectory model was selected as the final model, and the statistical parameters for the 2-, 3-, and 4-group trajectory models are shown in **Supplementary Table S1**. Based on model-adequacy criteria and interpretability, three distinct Ln(SIRI) trajectory groups were identified: low-stable (n = 2,095), middle-stable (n = 6,712), and high-stable (n = 2,816) (**Figure 3**). **Table 3** summarizes the baseline characteristics of these trajectory groups. Participants in higher Ln(SIRI) trajectory groups were more likely to be male and have higher rates of diabetes, hypertension, smoking, alcohol consumption, and higher levels of BMI, TG, SIRI index, SII index, PLR and NLR index (all *p* < 0.001). As Ln(SIRI) index trajectories increased, the risk of the progression of CAS increased (**Table 3**). These findings suggest a significant correlation between Ln(SIRI) trajectories and CAS progression. **Figure 4** presents the Kaplan-Meier survival curves for CAS progression by trajectory group (log-rank test, *p* < 0.001).

**Table 3:** Baseline characteristics of study participants according to trajectories of the Ln (SIRI) index.

Characteristic	Low-stable	Middle-stable	High-stable	P value
n	2095	6712	2816	
Age (years)	48 (40-55)	47 (39-54)	47 (39-54)	.075
Male, n (%)	1064 (50.8%)	4354 (64.9%)	2240 (79.5%)	.000
BMI (kg/m2)	23.5 (21.6-25.6)	24.1 (22.1-26.2)	24.66 (22.6-26.8)	.000
Diabetes mellitus, n (%)	35 (1.7%)	132 (2.0%)	86 (3.1%)	0.001
Hypertension, n (%)	68 (3.2%)	315 (4.7%)	183 (6.5%)	0.000
Alcohol consumption, n (%)	90 (4.3%)	486 (7.2%)	258 (9.2%)	0.000
Smoking, n (%)	177 (8.4%)	1047 (15.6%)	688 (24.4%)	0.000
H. Pylori positive, n (%)	544 (7.2%)	1891 (25.0%)	790(10.4&)	0.304
SBP (mmHg)	121 (111-132)	123 (113-135)	125 (115-137)	.000
DBP (mmHg)	73 (66-81)	75 (67-83)	76 (69-85)	.000
LDL (mmol/L)	2.63 (2.2-3.2)	2.60 (2.2-3.1)	2.58 (2.2-3.1)	0.061
HDL (mmol/L)	1.46 (1.3-1.7)	1.38 (1.2-1.6)	1.31 (1.2-1.5)	.000
TC (mmol/L)	5.05 (4.48-5.71)	4.95 (4.38-5.58)	4.91 (4.335-5.54)	.000
TG (mmol/L)	1.36 (0.4-2.1)	1.48 (1.0-2.3)	1.63 (1.1-2.5)	.000
FPG (mmol/L)	4.98 (4.7-5.3)	4.99 (4.7-5.3)	4.99 (4.6-5.4)	0.212
Leukocytes (1000 cell/mL)	5.2 (4.5-6.0)	6.1 (5.3-7.0)	7.2 (6.2-8.3)	.000
Neutrophil count (1000 cell/mL)	2.6 (2.2-3)	3.5 (2.9-4.1)	4.5 (3.8-5.3)	.000
Platelet count (1000 cell/mL)	223 (194-257)	236.5 (205-270)	244 (210-282)	.000
Lymphocyte count (1000 cell/mL)	2.1 (1.8-2.5)	2.1 (1.7-2.5)	2 (1.6-2.4)	.000
Monocyte count (1000 cell/mL)	0.3 (0.2-0.3)	0.3 (0.3-0.4)	0.4 (0.4-0.5)	.000
SIRI	0.3 (0.3-0.4)	0.6 (0.5-0.7)	1.0 (0.8-1.3)	.000
SII	274.8 (217.2-349.6)	394.1 (313.5-479.2)	548.3 (436.7-693.8)	.000
NLR	1.2 (1.0-1.5)	1.7 (1.4-2.0)	2.3 (1.9-2.8)	.000
PLR	106.0 (87.1-130.0)	115.0 (94.5-140.0)	122.5 (100.4-151.8)	.000
LMR	8 (6.7-9.5)	6 (5-7)	4.5 (3.8-5.3)	.000
CAS progression, n (%)	377 (18.0%)	1392 (20.7%)	691 (21.2%)	.000

### 3.4 Associations between Ln(SIRI) index trajectories and CAS progression

The association between Ln(SIRI) trajectory patterns and CAS progression is outlined in **Table 4**. When Compared to the low-stable group, both the middle-stable group and high-stable group demonstrated a higher likelihood of CAS progression. Following adjustments for covariates including age, FPG, SBP, and BMI, the high-stable group exhibited a 1.166-fold risk of CAS progression (HR = 1.166, 95% CI 1.021–1.333, *p* = 0.024). Additionally, the middle-stable group did not show a significant association with CAS progression (HR = 1.064, 95% CI 0.944–1.199, *p* = 0.310).

**Table 4:** Hazard ratios (95% confidence intervals) of CAS progression by trajectory groups of Ln (SIRI) index.

Ln (SIRI) index trajectories	CAS progression (N)	Unadjusted HR (95%CI)	P value	Model 1 HR (95%CI)	P value	Model 2 HR (95%CI)	P value
Low-stable	377/2095	Reference		Reference		Reference	
Middle-stable	1392/6712	1.111 (0.988-1.250)	0.079	1.078 (0.956-1.214)	0.220	1.064 (0.944-1.199)	0.310
High-stable	691/2816	1.305 (1.146-1.486)	<0.001	1.194 (1.046-1.363)	0.009	1.166 (1.021-1.333)	0.024

Model1: Adjusted for age, SBP and FPG;

Model2: Adjusted for age, SBP, FPG and BMI.

## 4 Discussion

In this large-scale longitudinal cohort study based on routine health examinations, we investigated the association between baseline SIRI levels, their long-term trajectories, and the progression of CAS. Elevated baseline SIRI values were significantly linked to CAS progression, whether analyzed as continuous variables or categorized into quartiles. Additionally, we identified three distinct SIRI trajectory patterns—low-stable, middle-stable, and high-stable—each associated with varying risks of CAS progression. Notably, the high-stable SIRI trajectory independently predicted CAS progression, even after accounting for baseline SIRI levels. These findings highlight the potential role of sustained systemic inflammation in driving the development and progression of CAS.

In recent years, the SIRI has acquired significant attention in the field of atherosclerotic cardiovascular disease (ASCVD) and coronary artery calcification. Dziedzic et al. revealed a positive correlation between the SIRI index and both the severity of coronary artery disease and the incidence of acute coronary syndrome (38). Hui Sun et al. further elucidated that elevated SIRI levels in patients with acute myocardial infarction (AMI) act as an independent risk factor, influencing the severity of coronary artery disease and holding predictive value (39). Tomasz Urbanowicz demonstrated that patients with an SIRI above 1.22 (area under the curve: 0.725, p < 0.001) had a significantly higher likelihood of developing single and complex coronary disease (40). Collectively, these studies underscore the independent association of the SIRI index with the incidence, development, progression, and adverse outcomes of ASCVD. Man Liao et al. reported significantly higher SIRI values in individuals with carotid atherosclerosis compared to those without, with logistic regression analysis corroborating the link between SIRI and carotid atherosclerosis (21). Our results are consistent with and significantly extend the growing body of evidence linking SIRI to cardiovascular disease. A recent retrospective cohort study by Nai et al (23). similarly found that a higher baseline SIRI was associated with an increased incidence of carotid plaque in a Chinese population free of baseline atherosclerosis. While their study established the prognostic value of a single SIRI measurement, our study advances this concept by demonstrating that tracking the trajectory of SIRI over time provides superior risk insight. We identified that individuals maintaining a high-stable SIRI pattern faced the greatest risk, suggesting that chronic, sustained inflammation is more deleterious than transient elevations. Furthermore, the association between SIRI and CAS appears robust across different patient populations. A cross-sectional study by Wang et al. in patients with chronic kidney disease (CKD) reported significant associations between SIRI and other novel inflammatory indices (e.g., SII, AISI, MHR) with the presence of carotid plaques. Their study importantly highlighted the partial mediating role of renal function (eGFR) in this relationship, illustrating the complex interplay between inflammation and end-organ damage in a high-risk cohort. Our study complements these findings by showing that SIRI remains a powerful predictor of CAS progression even in a general population without advanced CKD, indicating that its predictive value is not solely dependent on the backdrop of significant renal impairment (24).

Our baseline analysis revealed that participants with higher SIRI values were more likely to be male and, interestingly, tended to be younger. This observation is supported by previous studies and can be explained by several factors. The well-documented sexual dimorphism in immune response may account for the gender disparity, with males often exhibiting stronger innate immunity, while females typically mount a stronger adaptive immune response, influenced in part by the immunomodulatory effects of sex hormones like estrogen (41–43). The inverse association with age may initially seem paradoxical but likely reflects our study’s exclusion criteria. By excluding individuals with existing major diseases, we may have selected a cohort of healthier older adults with lower baseline inflammation (“healthy survivor effect”) (44). In this context, a high SIRI in a younger individual could be a particularly sensitive marker of pathological, premature inflammation, identifying a subgroup at heightened risk for future cardiovascular events (45, 46). This underscores the clinical utility of SIRI for early risk stratification.

The restricted cubic spline analysis revealed a complex non-linear relationship between SIRI and CAS progression risk. The risk increased progressively until reaching an inflection point at approximately SIRI = 0.35, beyond which the association plateaued. This plateauing effect may suggest a saturation phenomenon where extremely high levels of systemic inflammation do not confer additional risk, possibly due to immune exhaustion or competing risk factors. The point at which the hazard ratio crossed 1.0 was observed at SIRI = 0.5, providing a potential clinical threshold for risk stratification.

To ensure the study focused on chronic inflammatory status and existing atherosclerosis, we excluded individuals with potential acute infections, defined by leukocyte counts ≥14×10^9^/L. Unlike composite indices, individual blood cell counts are susceptible to variations caused by changes in fluid balance. In our study, individuals in the highest quartile of SIRI and the High-stable SIRI group often exhibited neutrophilia, monocytosis, and lymphocytopenia, indicating a combination of nonspecific inflammation and damage in the adaptive immune response (47). We propose that the interplay between these cellular changes creates a self-amplifying cycle of immune dysregulation that critically drives plaque progression and vulnerability. The observed monocytosis is particularly consequential in the context of established mechanisms of plaque infiltration. Circulating monocytes are heterogenous, and distinct subsets contribute differentially to atherogenesis (48). Classical monocytes (CD14^++^CD16^-^), which are likely predominant in our cohort, are rapidly recruited to sites of endothelial injury via interactions between CCR2 and its ligand MCP-1 (CCL2), which is highly expressed in inflamed vasculature (49). Upon entry into the plaque, they differentiate into inflammatory macrophages, extensively phagocytose oxidized lipids, and become foam cells—the hallmark of atheroma. Conversely, non-classical monocytes (CD14^+^CD16^+^) patrol the endothelium via CX3CR1 and may contribute to late-stage plaque progression through matrix metalloproteinase production, potentially undermining the fibrous cap’s stability (48, 50). The concomitant neutrophilia suggests an additional, potent driver of endothelial dysfunction. Activated neutrophils exacerbate vascular damage not only through degranulation but also via the release of neutrophil extracellular traps (NETs) (51, 52). NETs, web-like structures of chromatin and cytotoxic enzymes, directly inflict damage on endothelial cells, impairing their function and promoting a pro-thrombotic state (53). Furthermore, NETs can activate macrophages, prompting them to release potent pro-inflammatory cytokines such as IL-1β and IL-6, thereby intensifying the local inflammatory cascade within the plaque (54). This inflammatory milieu is further compounded by lymphocytopenia. The reduction in lymphocyte count, potentially driven by activation-induced apoptosis, signifies a loss of immunoregulatory control. A critical deficit in regulatory T cells (Tregs) diminishes a vital source of anti-inflammatory cytokines (e.g., IL-10 and TGF-β), allowing innate immune activation to proceed unchecked (55). Paradoxically, the apoptosis of lymphocytes itself may not be benign. The engulfment of apoptotic lymphocytes by macrophages can stimulate, rather than suppress, further pro-inflammatory cytokine production (e.g., TNF-α), creating a vicious cycle that perpetuates endothelial dysfunction and plaque growth (56, 57). Our findings suggest that SIRI integrates key mechanisms—NETs, foam cell formation, and immune dysregulation—into a single, clinically accessible metric. Targeting the interactions between these cell types may offer innovative therapeutic avenues for mitigating chronic inflammation in CAS.

The primary strength of this study lies in its large-scale, longitudinal, population-based cohort design, which included repeated assessments of SIRI and carotid ultrasound findings. The use of LCTM provided detailed insight into temporal changes in inflammatory activity. However, several limitations should be noted. First, the outcomes were qualitatively assessed; incorporating quantitative measures could improve the precision of analyses between SIRI levels and carotid intima-media thickness or plaque progression. Second, as a retrospective study, it is susceptible to certain biases. Third, diabetes was defined based on a single fasting blood glucose measurement (≥7.0 mmol/L) or self-reported physician diagnosis. Although this approach is common in large epidemiological studies (58), it deviates from standard clinical practice, which typically requires a second confirmatory test. This may have led to misclassification—for example, by including individuals with transient hyperglycemia.

## 5 Conclusions

This study revealed that individuals with a elevated baseline SIRI levels or a high-stable SIRI trajectory face a significantly higher risk of CAS progression. These findings underscore the importance of closely monitoring the SIRI index during regular health assessments to promptly identify the development of carotid atherosclerosis, thereby facilitating more effective prevention and treatment strategies.

## Data Availability

The raw data supporting the conclusions of this article will be made available by the authors, without undue reservation.

## Supplementary material

Supplementary Figure 1 Flowchart of the methodology for determining CAS progression.

Supplementary Figure 2 The restricted cubic spline was used to analyze the relationship between SIRI and CAS progression.
