# Supplementary figures and images for: Association between systemic inflammation response index trajectories and carotid atherosclerosis progression
Source: Front Endocrinol (Lausanne). 2025 Oct 14;16:1676493. doi: 10.3389/fendo.2025.1676493 (PMC12558823; doi:10.3389/fendo.2025.1676493)

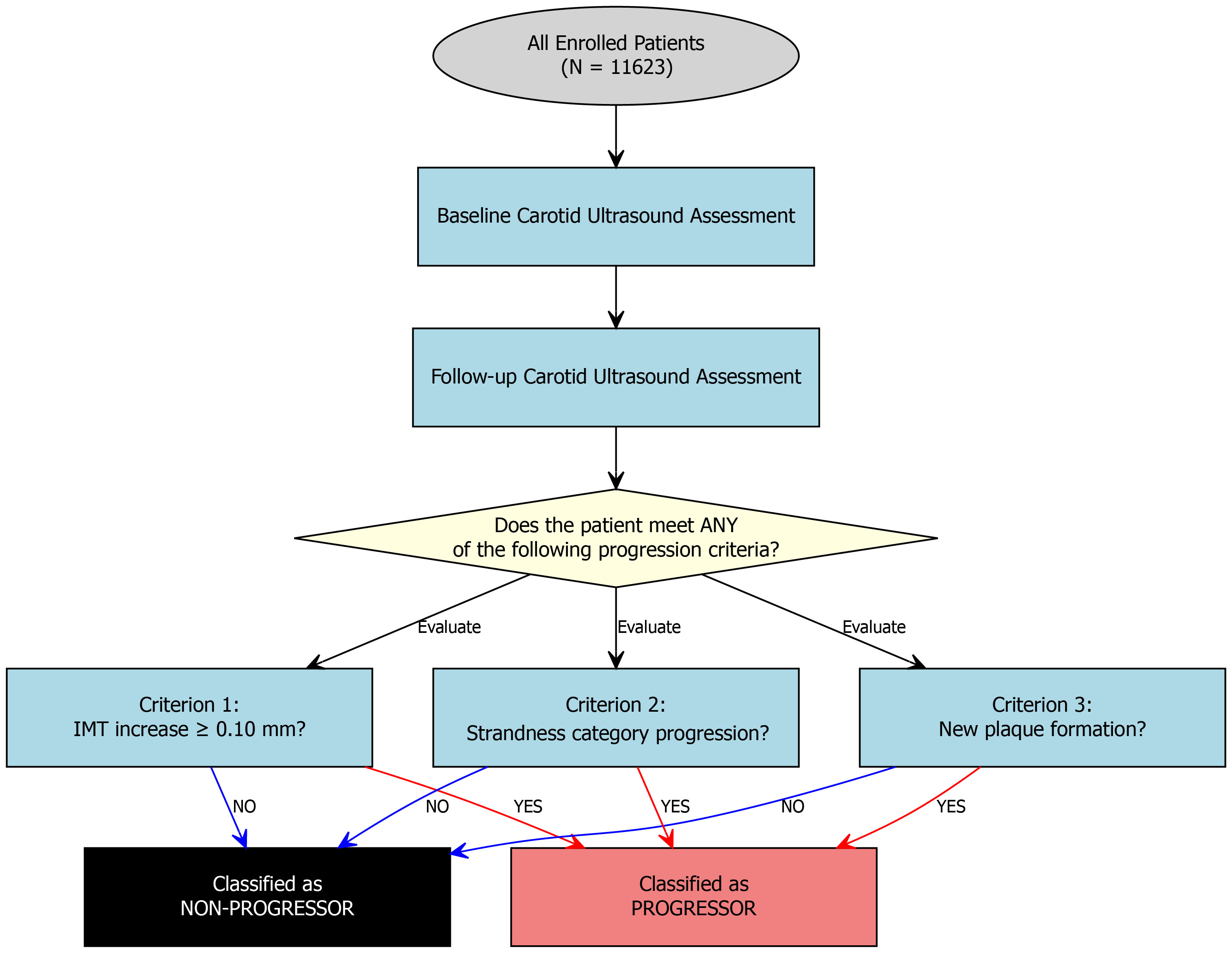

Supplement: Supplementary Figure 1 — Flowchart of the methodology for determining CAS progression. [file Image1.jpeg]

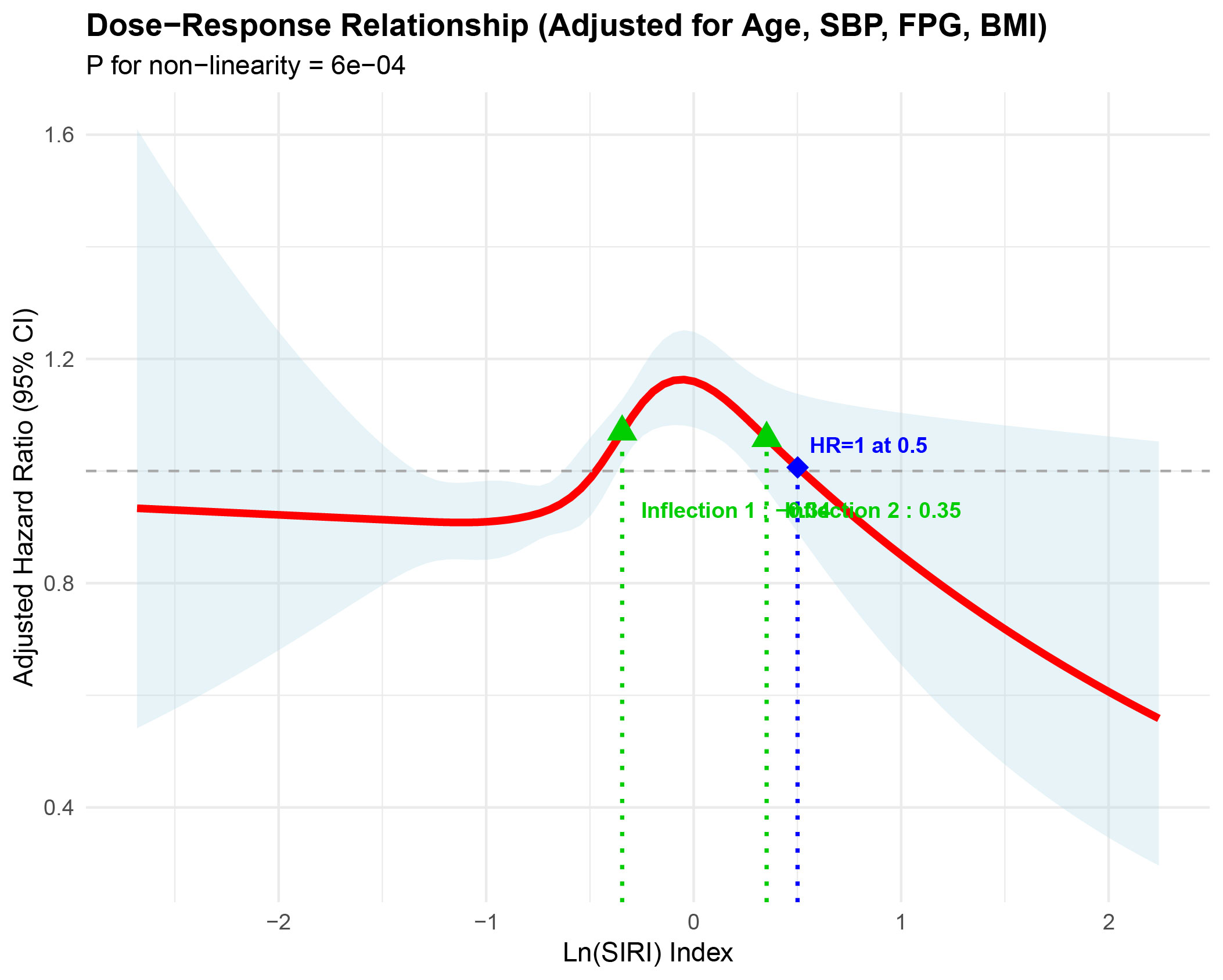

Supplement: Supplementary Figure 2 — The restricted cubic spline was used to analyze the relationship between SIRI and CAS progression. [file Image2.jpeg]
