# Supplementary material for: Association between systemic inflammation response index trajectories and carotid atherosclerosis progression
Source: Front Endocrinol (Lausanne). 2025 Oct 14;16:1676493. doi: 10.3389/fendo.2025.1676493 (PMC12558823; doi:10.3389/fendo.2025.1676493)
Supplement: Supplementary file 3 [file Table1.docx]

**Table S1. Statistical parameters for 2-, 3-, and 4-** **latent class trajectory modeling (n = 11623).**

| **Parameter** | **Number of groups** | | |
| --- | --- | --- | --- |
|  | **2** | **3** | **4** |
| **BIC** | -32492.64 | **-30775.36** | -30094.68 |
| ***N* (observed group proportion, %)** | | | |
| Group 1 | 5323 (45.8) | **2095 (19.4)** | 942 (8.1) |
| Group 2 | 6300 (54.2) | **6712 (55.1)** | 4382 (37.7) |
| Group 3 |  | **2816 (25.5)** | 5044 (43.4) |
| Group 4 |  |  | 1255 (10.8) |
| **AvePP** | | | |
| Group 1 | 0.904 | **0.86** | 0.85 |
| Group 2 | 0.91 | **0.85** | 0.82 |
| Group 3 |  | **0.86** | 0.81 |
| Group 4 |  |  | 0.83 |
| **Entropy** | 0.69 | **0.70** | 0.70 |

Note: The trajectory of each group was the quartic polynomial order.

BIC: Bayesian information criterion; AIC: Akaike Information Criterion; AvePP: Average posterior probability.
